# Comparative Analysis of the Influence of Chemical Composition and Microstructure on the Abrasive Wear of High-Strength Steels

**DOI:** 10.3390/ma15145083

**Published:** 2022-07-21

**Authors:** Martyna Zemlik, Łukasz Konat, Jerzy Napiórkowski

**Affiliations:** 1 The Faculty of Mechanical Engineering, Department of Vehicle Engineering, Wroclaw University of Science and Technology, 50-370 Wrocław, Poland; lukasz.konat@pwr.edu.pl; 2 The Faculty of Technical Sciences, Department of Building and Exploitation of Vehicles and Machines, University of Warmia and Mazury in Olsztyn, 10-719 Olsztyn, Poland; napj@uwm.edu.pl

**Keywords:** martensitic steels, boron steels, abrasive wear resistance, microstructure, prior austenite grain size, XAR, TBL, Creusabro

## Abstract

The paper discusses the microstructural, chemical and tribological properties of the selected low-alloy, high-strength martensitic boron steels with a hardness of 500–600 HBW. These materials, due to their increased strength, and thus resistance to abrasive wear, are widely used in the mining, agricultural or building industries. Grades such as XAR, TBL and Creusabro were subjected to a comparative analysis. As a result of the conducted research, an attempt was made to determine the relation between the microstructural properties, chemical composition, hardness and abrasive wear resistance of the above-mentioned metallic materials belonging to the same material group. The scope of work involved a metallographic analysis, including the examination of the microstructure with an analysis of the prior austenite grain size. Tribological tests were carried out with the use of a T-07 tester, which is designed for testing abrasive wear resistance in the presence of a loose abrasive. As a result, it was found that the coefficient of relative abrasion resistance k_bAV_ in relation to as-normalized C45 steel is equal to 0.9–1.25 and may even have the same value among materials of different hardness in the as-delivered state.

## 1. Introduction

The materials used for components that are subjected to abrasive wear can be divided into five groups, i.e., chromium cast iron, Hadfield cast steel, hardfaced materials, cemented carbides and martensitic steels. The last group includes grades such as Hardox, Raex, XAR, Miilux, Relia, Creusabro, Abrazo, Endura or HTK. They are most often classified according to hardness measured in the Brinell scale, with values being approximately equal to 400, 450, 500, 550 and 600 HBW. An innovation in this area turned out to be the production of Hardox Extreme steel, which has a hardness of over 600 HBW and a tensile strength significantly exceeding 2000 MPa (average 2461 MPa [1]). Obtaining a uniform martensitic microstructure across the entire cross-section is a result of the use of alloying micro-additives that act as hardenability enhancers. Examples of such additives include chromium, molybdenum and manganese. However, in this respect, the boron micro-additive deserves particular attention. Its influence on hardenability is, among other things, attributed to the segregation mechanism at the austenite grain boundaries, due to which phase transformation is delayed. The maximum permissible boron concentration is 0.0025% by weight, and exceeding this limit causes the coagulation of Fe_2_B borides, which are the preferred nuclei in the growth process of ferrite grains. In addition, boron has a strong affinity for nitrogen, and therefore titanium and aluminum micro-additives are used to prevent the release of boron nitrides or oxides, as well as to ensure that an adequate amount of this element is dissolved in the matrix.

The appropriate selection of a material for components that are susceptible to abrasive wear, e.g., ploughshares, cultivator teeth, bucket wheel chutes or excavator buckets parts, should take into account an analysis of operating conditions, including the type and way of transferring loads, as well as an assessment of technological and constructional feasibility. Moreover, such elements are often exposed to dynamic loads and should therefore also show resistance to impact wear. Meeting these requirements is possible by using martensitic steels, which are characterized by both high strength indices and satisfactory plastic properties. These features are the result of the low content of harmful elements (P and S), the fine-grained structure and the manufacturing method that uses thermomechanical rolling in the steel mill. What is more, these materials can also be supplied in a softened state, with their heat treatment then being performed on profiled sections (TBL steels, B27 and Miilux boron steel).

The assessment of abrasive wear resistance is complex and cannot be interpreted solely as a function of the amount of weight loss. Although the available research indicates that resistance to abrasive wear is proportional to the hardness of the material [2,3,4,5], the above correlation is not valid if the components are additionally subjected to impact wear. On the basis of the results of tests carried out with the use of an impeller-tumbler device, which is designed to simulate the above-mentioned working conditions, it may be indicated that only materials of an analogous structure show a linear relation in this respect [2,6,7,8]. It should be noted here that steels are generally characterized by a decrease in impact strength and ductility with an increase in static strength. However, according to [8], steel with a nominal hardness of 650 HB showed the highest impact wear resistance, and it can thus be assumed that these properties remain satisfactory. The above conclusions were also confirmed in [9], where Hardox 500 steel plates applied to a bucket wheel chute showed no pronounced wear when compared to 18G2A steel (P355N) hardfaced with an Fe-Cr-C alloy. Moreover, this statement goes against the results of the tribological analysis performed using a T-07 device, which enables wear resistance in the presence of a loose abrasive to be tested. It is estimated that 60% of wear loss in mining is due to the abrasive wear of critical components [10]. Materials science research, conducted in the course of field tests (during exploitation) and applied to determine the appropriateness of using a given material on a particular component, require significant time and money. Due to this, laboratory tests are often used to determine the correlation with actual operating conditions. In this regard, the use of the Holm–Archard relation (1), applied to describe wear during sliding friction, shows satisfactory adaptability [11]. Moreover, a laboratory analysis using the T-07 device can be comparable with field tests of ploughshares [12].

(1) $$I_{Z}=\frac{k\times P\times l}{3H}$$

where:

*I_z_*—rate of wear (m^3^);
*k*—wear coefficient;
*P*—force (N);
*l*—friction distance (m);
*H*—hardness (N/mm^2^).

The mechanism of abrasive wear itself can be divided into microploughing, which is associated with the plastic deformation of the material, microcutting, in which there is a reduction in mass due to the cutting action, and also scratching and fatigue wear. The nature of wear depends on the microstructure of the material, and the material’s hardness and fracture toughness. Additional factors influencing the degree of wear are microstructural parameters such as the grain size of the prior austenite. For example, according to [13], the abrasive wear resistance of Hardox 450 steel decreases as the grain size increases above 40 µm. Moreover, in [14], it was shown that steel with a nominal hardness of 500 HBW has more favorable tribological wear indices when its microstructure consists of equiaxial grains of about the size of 14 µm. The grain growth of the former austenite mainly causes a decrease in the impact strength [15,16]. The above microstructural relationships also impinge on the work-hardening ability of the sub-surface layer [17], with the discussed features possibly influencing different abrasive wear characteristics—even among materials of the same hardness in the as-delivered state [18,19,20,21]. The above mechanisms have not yet been explored, and steel producers and customers may not be aware of the different tribological properties between steel grades offered for the same purpose.

**Figure 1:**
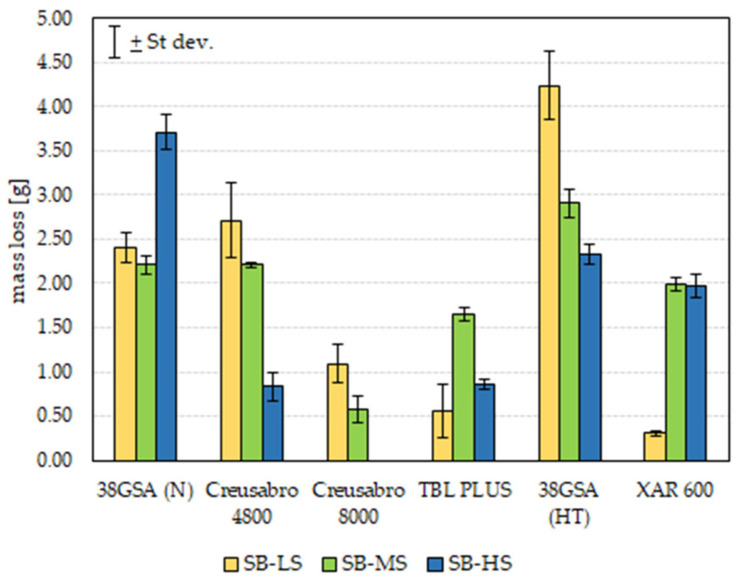
The mass wear of the tested samples over a friction distance of 20,000 m in various kinds of abrasive soil masses. SB-LS—“spinning bowl” method, light soil; SB-MS—“spinning bowl” method, medium soil; SB-HS—“spinning bowl” method, heavy soil; N—as-normalized condition, HT—as-hardened condition [22,23,24].

**Figure 2:**
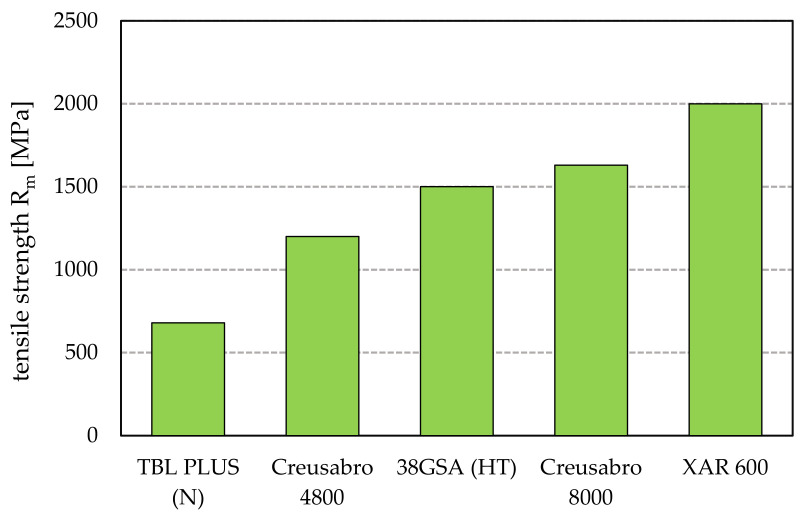
Tensile strength R_m_ of the tested types of steels based on manufacturer’s data. N—as-normalized condition, HT—as-hardened condition [25,26,27,28,29].

Therefore, the aim of this paper is to indicate the microstructural differences between exemplary low-alloy martensitic steels, with a declared hardness of 500–600 HBW, and their tribological properties under abrasive wear conditions. The steel analyzed was 38GSA (38MnSi4), which, despite no longer being produced, is still commercially available and used for ploughshares. The other grades were TBL PLUS and XAR 600, as well as Creusabro 4800 and 8000. Creusabro steels, according to the manufacturer’s data, are characterized by a complex bainitic-martensitic microstructure with finely dispersed carbides and residual austenite. Their resistance to abrasive wear is determined primarily by the ability of the sub-surface layer to work, hardening as a result of the TRIP effect (transformation-induced plasticity), which consists of the formation of martensite from metastable austenite by its deformation. The above phenomenon is the reason for the high wear resistance exhibited by Hadfield steels. TBL PLUS steel is a material sold in the as-normalized condition, with its main purpose being its use in the working parts of agricultural machinery. The presented analysis is a continuation of the research conducted in [22,23,24], where, despite similar microstructural and mechanical properties such as hardness and tensile strength, varied and non-linear results were observed during wear resistance tests in different abrasive soil masses, i.e., light, medium and heavy soils (Figure 1). The texture of soil (its heaviness to be cultivated) characterizes the resistance that agricultural machinery encounters during work. It defines the content of different granulometric fractions (sand, silt and clay) and, depending on their ratio, the soil is characterized by a different agronomic category. Light soil has a low clay content and consists mainly of sand with a particle size up to 2 mm, and it is therefore free-flowing. Heavy soil, on the other hand, is compact, with its main component involving clay with a grain size < 0.002 mm. The mechanical properties declared by the manufacturers of the steels in question are shown in Figure 2. The similar mechanical properties of the 38GSA and Creusabro 8000 steels do not translate into a linear increase in abrasive wear resistance—in the case of 38GSA, the weight loss is up to four times higher in light soil. Moreover, XAR 600 steel, with a claimed hardness of >550 HBW, shows lower wear resistance when tested in a medium-to-heavy soil mass compared to the less durable TBL PLUS and Creusabro steels. These results motivated the authors to determine the basic parameters of the analyzed steels. The rationale behind this approach was the fact that the TBL PLUS, XAR 600 and 38GSA steels have a similar carbon content, which in turn means that the analyzed steels have similar tribological properties in analogous working conditions.

## 2. Methodology

For this study, sheet sections with thicknesses of 10 mm (TBL PLUS, XAR 600, 38GSA) and 15 mm (Creusabro 4800 and 8000) were used. They were cut out using a high-energy abrasive water jet—a technology that ensures the preservation of the microstructure formed at the stage of metallurgical processing. The sheets were supplied directly by the manufacturer or by an authorized distributor (STAL-HURT, Marciszów, Poland).

The TBL PLUS steel was delivered in the as-hardened condition. The 38GSA steel was subjected to heat treatment that included quenching in water from 880 °C after austenitizing for 20 min. For soaking, a Czylok FCF 12SHM/R gas-tight chamber furnace (Jastrzębie-Zdrój, Poland) with a 99.95% argon protective atmosphere was used.

Metallographic studies were carried out by means of a Nikon Eclipse MA200 light microscope (Tokyo, Japan) at magnifications in the range of 25–500×. The material was etched with HNO_3_ solution according to PN-H-04503:1961P. Images of the microstructures were captured with a Nikon DS-Fi2 digital camera, while their analysis was performed using NIS Elements 4.13.03 software (Nikon Corporation, Tokyo, Japan).

Chemical analysis was carried out on the cross-section of the analyzed sheets by means of a GDS500A Leco (Saint Joseph, MI, USA) glow discharge–atomic emission spectrometer with the following parameters: U = 1250 V, I = 45 mA, 99.999% argon, where the results were arithmetic averages of five measurements.

Brinell hardness was measured using a Zwick/Roel ZHU 187.5 universal tester (Ulm, Germany) at 187.5 kgF (1838.7469 N) according to EN ISO 6506-1:2014-12. The diameter of the used hardmetal ball was 2.5 mm.

To reveal the grain boundaries of the former austenite, the material was tempered at 550 °C for 30 min and then cooled with a furnace. Mi7Fe reagent (2 g picric acid, 1 g of sodium alkylsulfonate, 100 mL H_2_O), according to ASTM E407, was used for etching. Measurement of the grain size was performed according to PN-EN ISO 643:2020-07 by means of ImageJ ver. 1.52a software. The obtained results are the arithmetic average from 100 measurements.

**Figure 3:**
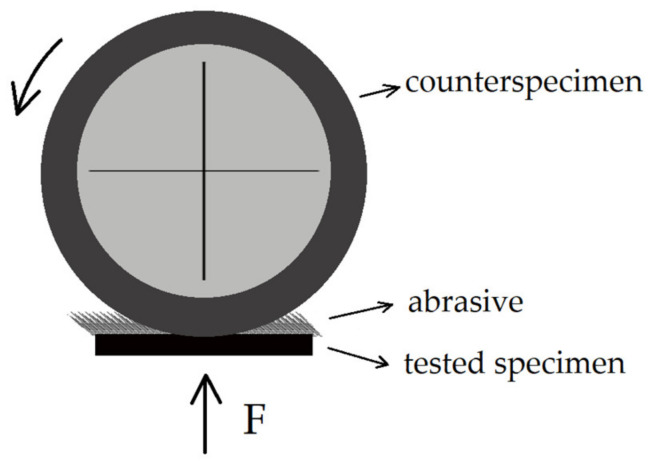
The scheme showing the principle of work of the T-07 tester.

Examinations of abrasive wear resistance were performed using a T-07 tester (Institute for Sustainable Technologies—National Research Institute in Radom, Poland), acc. to GOST 23.208-79, under a constant load of F = 44 N (ΔF = 0.25 N). The difference between the T-07 tester and the tribotester described in international standard ASTM G65 consists of the way to locate the examined material. For the T-07 tester, the specimen is placed horizontally, and for the tribotester described in ASTM—vertically. During examination, specimens with dimensions of 30 × 30 × 3 mm, made of the research and the reference materials, were subjected to wear with abrasive particles introduced to the friction contact zone. As an abrasive, aloxite no. 90, acc. to ISO 8486-2:1998, was used, and the reference specimen was made of C45 steel in the as-normalized condition. The duration of the test was selected with regards to the material hardness and was equal to 30 min (1800 revolutions of the roll). The coefficient k_bAV_, calculated according to Equation (2), was used as a measure of abrasive wear resistance. The principle of work of the tester is shown in Figure 3.

Images of the microstructures and surface topographies of the worn surfaces were recorded by means of a Phenom XL electron microscope (Phenom-World, Eindhoven, The Netherlands), which applied BSE imaging and an accelerating voltage of 15 keV.

(2) $$k_{b}=\frac{Z_{ww}\rho_{b}N_{b}}{Z_{wb}\rho_{w}N_{w}}$$

where:

*k_b_*—coefficient of relative abrasion resistance (dimensionless);
*Z_ww_*—mass consumption of the standard sample (g);
*Z_wb_*—mass consumption of the tested sample (g);
*N_w_*—number of rotations of the rubber-rimmed steel wheel during the test of the standard sample;
*N_b_*—number of rotations of the rubber-rimmed steel wheel during the test of the tested sample;
*ρ_w_, ρ_b_*—material density of the standard sample and tested sample (g/cm^3^).

## 3. Results

### 3.1. Chemical and Microstructural Analysis

Table 1 shows the chemical compositions and hardness measurements of the analyzed steels, according to the manufacturer’s data and based on the authors’ tests, respectively. The carbon contents of the TBL PLUS, XAR 600 and 38GSA steels are similar to each other (at 0.34–0.37%wt.), classifying them as medium carbon steels. As an exception, only the Creusabro steels (especially 4800) can be identified as low-carbon steels, having a carbon content of 0.2% for Creusabro 4800 and 0.27% for Creusabro 8000. In all these materials, manganese is the main hardenability enhancer, but it still has a strong effect on lowering the martensite start temperature M_s_. Therefore, its content is lowered in XAR 600 steels and compensated by the higher amount of other alloying elements that retard the diffusional transformation, i.e., chromium, molybdenum and nickel. Moreover, the addition of the latter lowers both the austenitizing temperature and the plastic–brittle transition temperature. Boron is present in the Creusabro 8000 and XAR 600 steels in the maximum allowable concentration. Higher amounts (as in the TBL PLUS steels) can cause the coagulation of Fe_2_B compounds, thus providing a favoured nucleation mechanism for inducing the growth of ferrite grains. In the Creusabro 4800 and 38GSA steels, the presence of boron was not demonstrated. The molybdenum content of 0.15 and 0.23% in the XAR 600 and Creusabro 8000 steels, respectively, justifies the possibility of carrying out tempering processes, in turn neutralizing the negative effect of chromium on tempering embrittlement. There is also a significant amount of silicon in the Creusabro 8000 and 38GSA steels, which, despite its beneficial effect on tempering processes after hardening, significantly reduces its impact toughness. It is also possible in all grades to distinguish trace amounts of titanium and aluminum. Boron, having a strong affinity for oxygen and nitrogen, reacts with these elements to form boron nitrides or oxides. Titanium and aluminum additives bind these gases into non-metallic phases so that an appropriate amount of boron remains dissolved in the matrix, which ensures the high hardenability of the material. Moreover, these compounds, which form barriers to dislocation movement, block the austenite grain growth. In all the analyzed steels, the content of the impurity admixtures (P and S) is negligible, thereby ensuring the preservation of high strength and plastic indices. Based on the results of the hardness measurements, it can be generally concluded that XAR 600 and 38GSA are steels of class 600 (HBW), while Creusabro 8000 and TBL PLUS represent class 500 (HBW).

**Figure 4:**
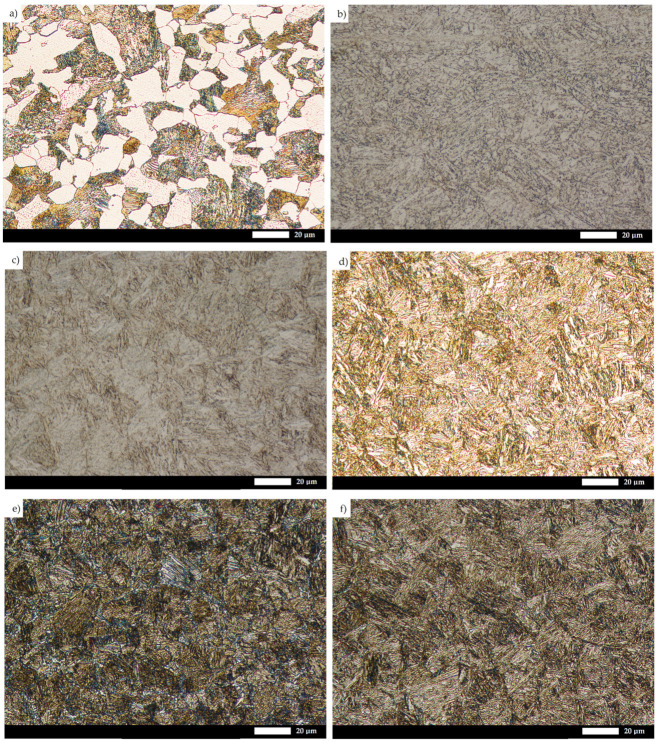
Microstructure of the tested types of steels: (**a**) 38GSA (N); (**b**) Creusabro 4800; (**c**) Creusabro 8000; (**d**) TBL PLUS; (**e**) XAR 600; (**f**) 38GSA (HT). Light microscopy, 500×, etched with 5% HNO_3_.

**Figure 5:**
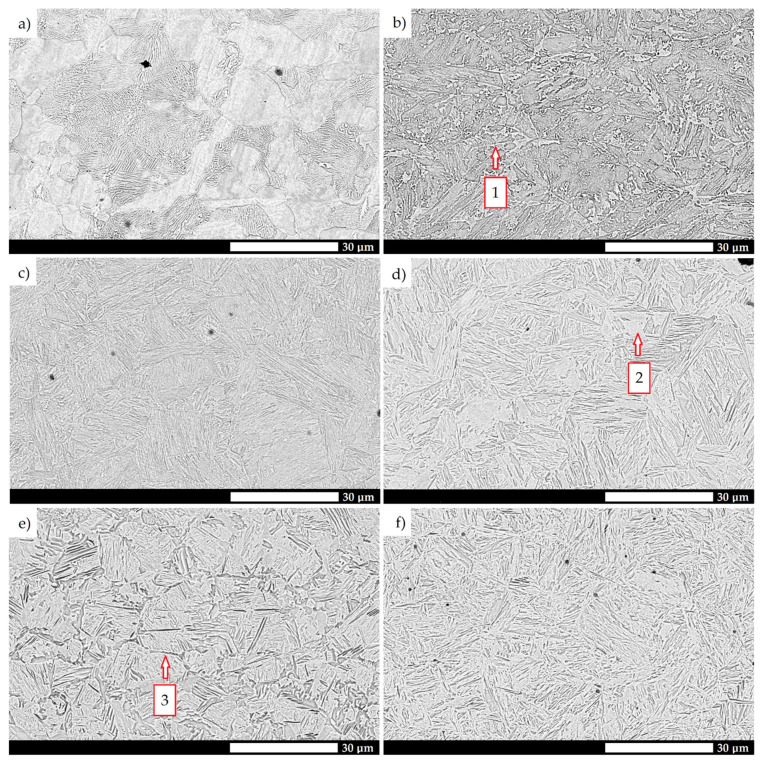
Microstructure of the tested types of steels: (**a**) 38GSA (N); (**b**) Creusabro 4800; (**c**) Creusabro 8000; (**d**) TBL PLUS; (**e**) XAR 600; (**f**) 38GSA (HT). 1—network of carbides distributed at the grain boundaries of the prior austenite, 2—untempered martensite, 3—non-martensitic phases at the grain boundaries of the prior austenite. SEM, etched with 5% HNO_3_.

A detailed microstructural analysis provides additional information on the differences between the analyzed materials (Figure 4 and Figure 5). Steel 38GSA is characterized by a ferritic-pearlitic microstructure with a slight banded structure in the as-normalized state. In addition, the pearlite colonies are varied in terms of lamellae thickness, and locally, areas of quenching troostite can be observed. Creusabro 4800 is characterized by a microstructure of tempering sorbite. Specific heat treatment procedures led to the coagulation of carbide phases in this steel, with the post-martensitic orientation still being preserved. It is also observable that carbides are distributed mainly at the grain boundaries of the former austenite, forming a characteristic continuous network. The microstructure of the Creusabro 8000 steel consists of both tempering sorbite and lath-tempering martensite. When compared to Creusabro 4800, it is characterized by a significantly smaller size of packets and blocks, with no significant amount of carbide precipitates being observed at the grain boundaries of the former austenite, especially in the form of a continuous network.

Microstructural differences are also observed between the TBL PLUS, XAR 600 and 38GSA steels. Although all of them are characterized by a lath martensite morphology, distinct properties can be pointed out, among others, in terms of the packet and block sizes or the occurrence of non-martensitic phases. The microstructure of the TBL PLUS steel is additionally composed of areas of untempered martensite, which is characterized by a high hardness, susceptibility to brittle fracture and reduced resistance to fatigue wear. A martensitic microstructure with clearly defined grain boundaries of former austenite is observed in the XAR 600 steel. Comparing the 38GSA steel to the XAR 600 and TBL PLUS steels, it can be seen that the 38GSA steel has the largest packet size. Furthermore, no carbide precipitates are observed in either of these steels, which may indicate that tempering operations were omitted in the production process.

**Figure 6:**
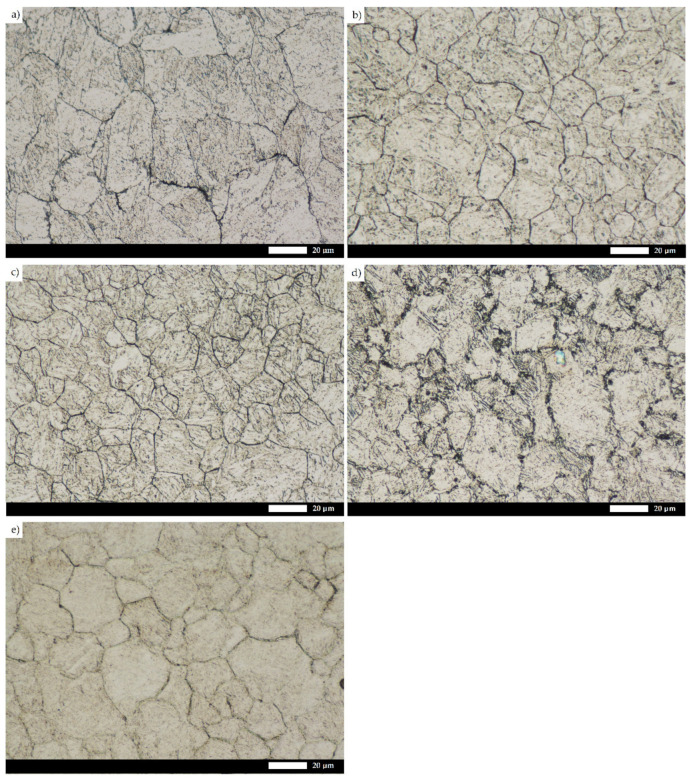
Microstructure of the tested types of steels with visible prior austenite grain boundaries: (**a**) Creusabro 4800; (**b**) Creusabro 8000; (**c**) TBL PLUS; (**d**) XAR 600; (**e**) 38GSA. Light microscopy, 500×, etched with Mi17Fe.

**Figure 7:**
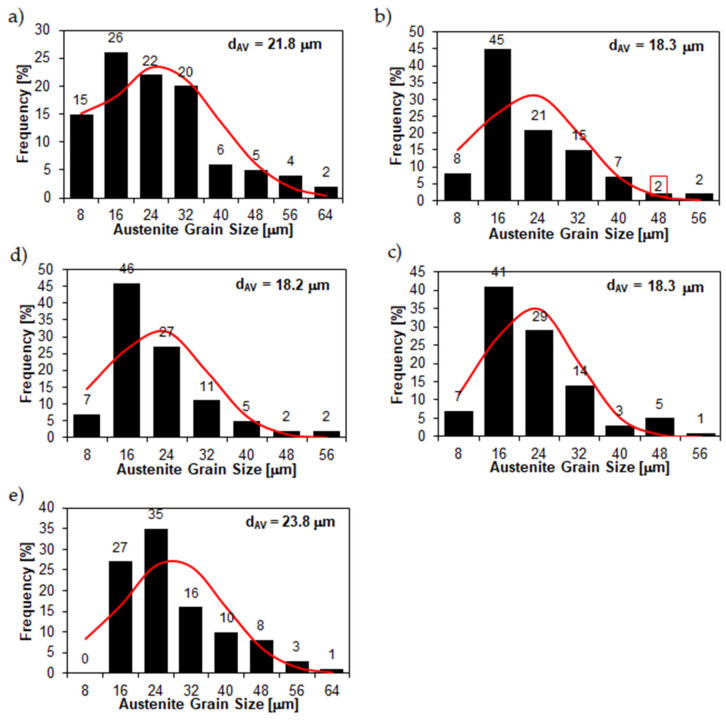
Frequency intervals and the normal occurrence distributions of the determined grain sizes of the prior austenite in the tested types of steels: (**a**) Creusabro 4800; (**b**) Creusabro 8000; (**c**) TBL PLUS; (**d**) XAR 600; (**e**) 38GSA; d_AV_—average grain diameter.

The microstructural results presented above are also confirmed by the grain size measurements of the prior austenite (Figure 6 and Figure 7), with the 38GSA steel having the largest grain size (average grain diameter d_AV_ = 23.8 µm). A slightly smaller size is shown by the Creusabro 4800 steel (d_AV_ = 21.8 µm), while the Creusabro 8000, TBL PLUS and XAR 600 steels have values analogous to each other, equal to 18.2–18.3 µm, allowing these materials to be classified as fine-grained. As such, the above variable of microstructural features can be excluded as an additional factor influencing the degree of wear.

These statements are reasonable due to their effects on the mechanical and plastic properties of the material, as well as on the abrasive wear resistance. According to the Hall–Petch relation, a smaller grain size increases the yield strength of the material and, in many cases, the static tensile strength. At the same time, a fine-grained microstructure also has a positive effect on the fracture toughness of the steel. According to [15], the grain growth in Hardox 450 steel up to 123.7 µm causes a decrease in the impact toughness from KCV = 70.3 J/cm^2^ to KCV = 19.0 J/cm^2^. It must be acknowledged that the above mechanical properties are crucial and, under operating conditions, affect the service life of such elements as chutes, hoppers and shovels, which are exposed to dynamic loads due to the impact of large, excavated masses. Another example is ploughshares, which are in direct-impact contact with large soil mass fractions or rock minerals present in the soil. The grain size of the former austenite impinges on the size of the packets and blocks of the martensitic microstructure, thus providing additional obstacles to dislocation movement. A smaller grain size results in higher fracture toughness due to energy loss during a change of propagation direction. Intermetallic phases such as nitrides, carbide nitrides and carbides, which block grain growth up to 1000 °C, help to preserve these properties. Based on microphotographs of the microstructure of the XAR 600 steel (Figure 6d), it can also be confirmed that the steel is resistant to tempering as it does not have a clearly visible network of carbide phases at the grain boundaries.

### 3.2. Analysis of Abrasive Wear Resistance

**Figure 8:**
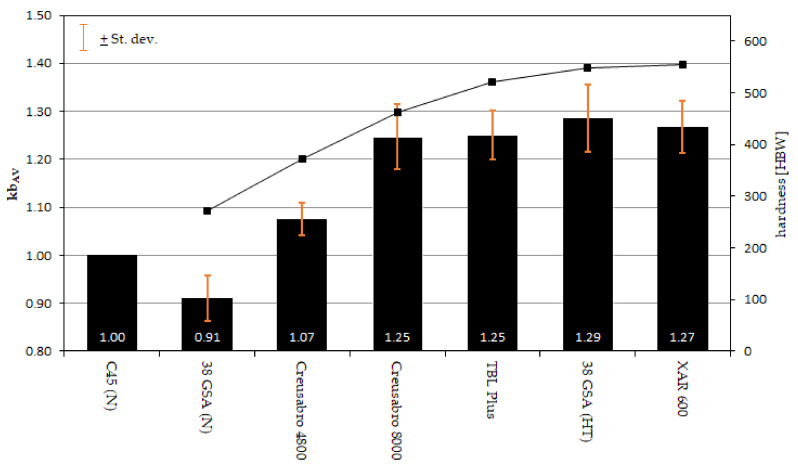
Coefficient of the relative abrasion resistance k_bAV_ of the analyzed types of steels with respect to hardness and prior austenite grain size.

In the course of the tribological tests, involving the assessment of resistance to abrasive wear in the presence of a loose abrasive, no significant differences were demonstrated between the Creusabro 8000, TBL PLUS, 38GSA (HT) and XAR 600 steels (Figure 8, Table 2). Their coefficient of relative abrasion resistance k_bAV_ with respect to C45 steel in the as-normalized condition is at the level of 1.25–1.29, and therefore the resistance of these steels can be considered to be very similar to each other. In comparison, according to [9], the values obtained are higher than the resistance of Brinar 500 and Hardox 500 (k_bAV_ equal to 1.11 and 1.20, respectively). Moreover, the study did not show a linear relationship between the hardness of the material and the abrasive wear resistance, and such a relationship could not be shown for the grain size of the former austenite either. However, the greatest grain size in the 38GSA steel can justify its similar abrasion resistance in comparison to the less hard TBL PLUS and Creusabro 8000 steels. The above phenomenon may be explained by the solid-solution strengthening and work-hardening ability of the sub-surface layer, analyzed further below.

**Figure 9:**
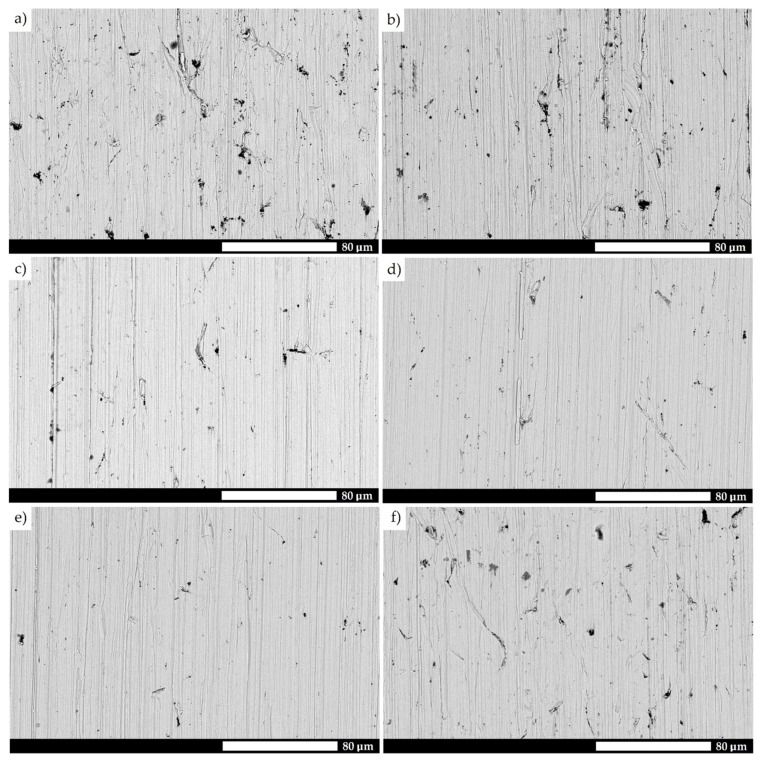
Images of worn surfaces. (**a**) 38GSA (N); (**b**) Creusabro 4800; (**c**) Creusabro 8000; (**d**) TBL PLUS; (**e**) XAR 600; (**f**) 38GSA (HT). SEM, unetched.

**Figure 10:**
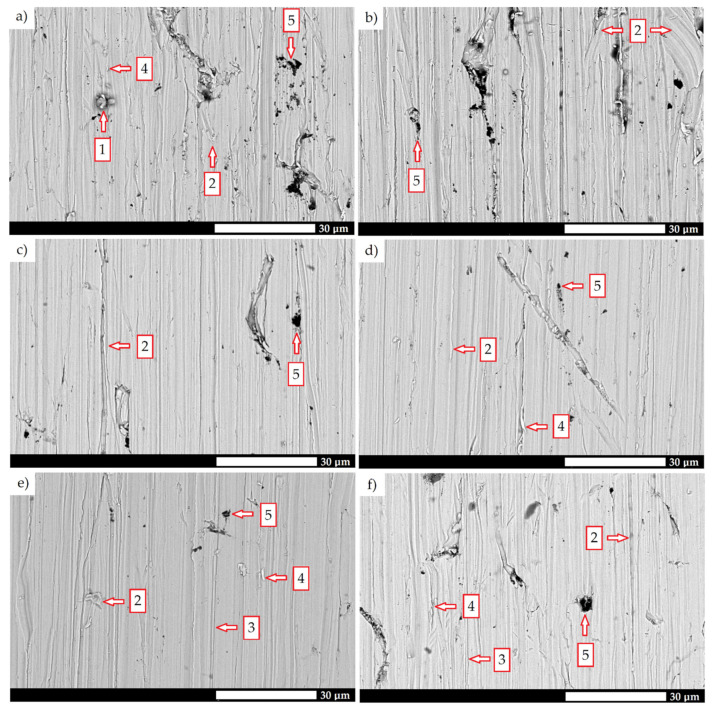
Images of worn surfaces. (**a**) 38GSA (N); (**b**) Creusabro 4800; (**c**) Creusabro 8000; (**d**) TBL PLUS; (**e**) XAR 600; (**f**) 38GSA (HT). 1—abrasive particle, 2—microploughing (furrows), 3—microcutting, 4—wear debris, 5—detached material. SEM, unetched.

**Figure 11:**
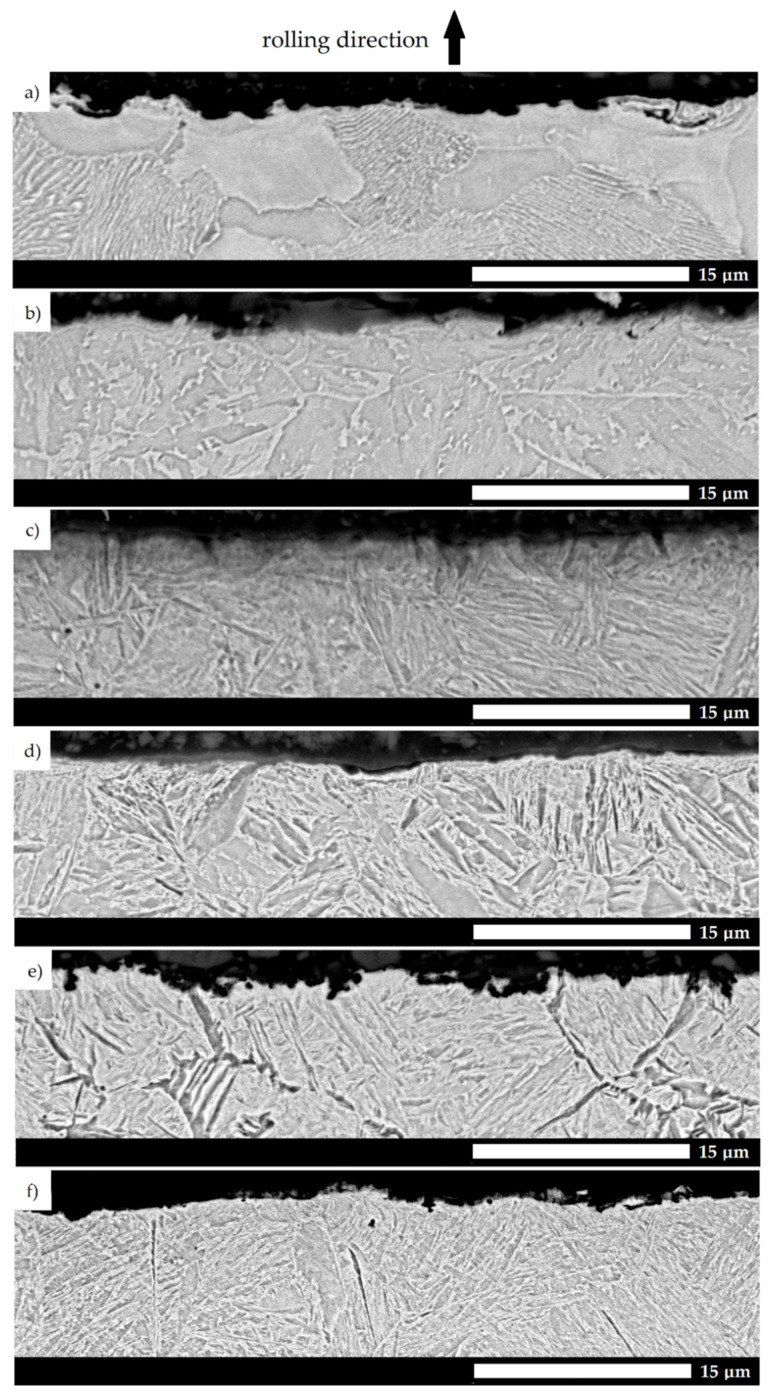
Sub-surface microstructures taken transverse to the direction of abrasive action. (**a**) 38GSA (N); (**b**) Creusabro 4800; (**c**) Creusabro 8000; (**d**) TBL PLUS; (**e**) XAR 600; (**f**) 38GSA (HT). SEM, etched with 5%HNO_3_.

**Figure 12:**
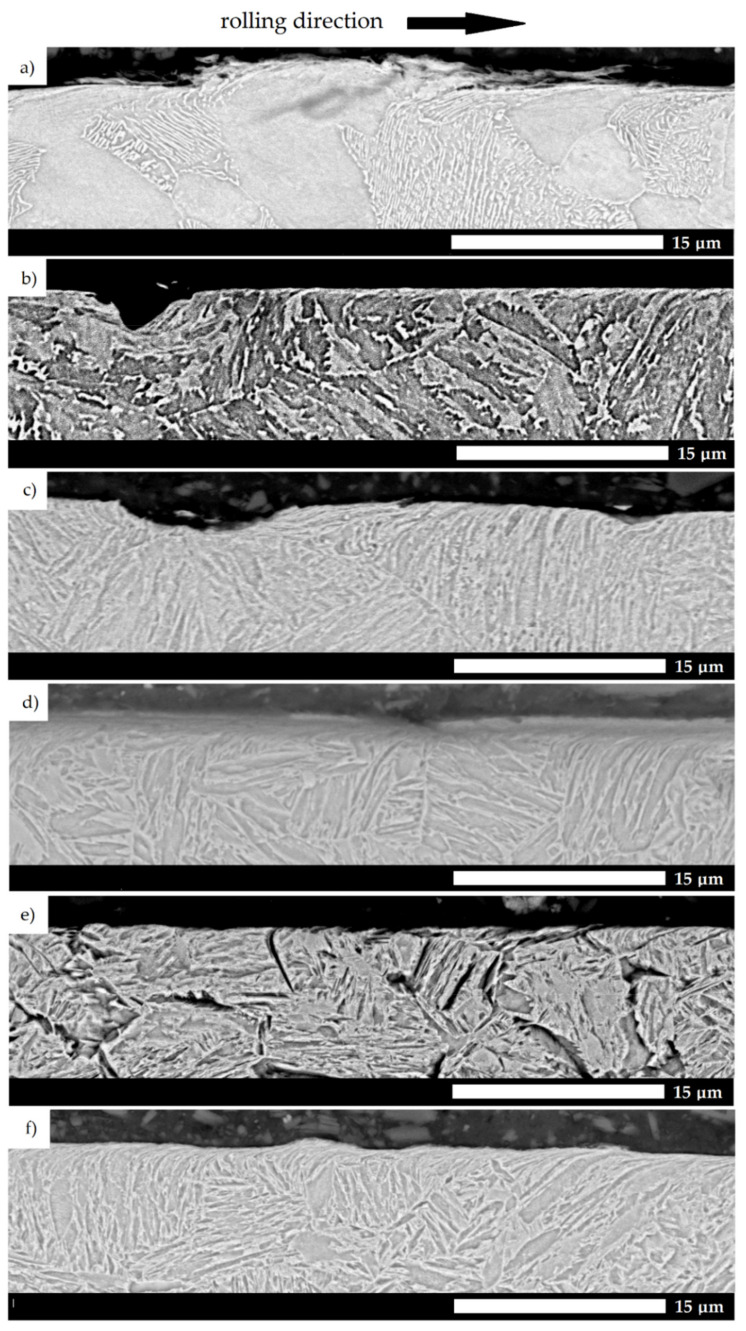
Sub-surface microstructures taken longitudinal to the direction of abrasive action. (**a**) 38GSA (N); (**b**) Creusabro 4800; (**c**) Creusabro 8000; (**d**) TBL PLUS; (**e**) XAR 600; (**f**) 38GSA (HT). SEM, etched with 5% HNO_3_.

More detailed information on the mechanisms of the abrasive wear of the steels under consideration is provided by the evaluation of the surfaces subjected to tribological tests (Figure 9 and Figure 10), as well as the surface topographies of the cross-sections taken in both the transverse (Figure 11) and longitudinal (Figure 12) direction of the abrasive action. According to Figure 9, the frequency of the occurrence of significant tribological changes is the most intensive in the 38GSA (both heat treatment states) and Creusabro 4800 steels. The marks on the surface of the as-normalized 38GSA steel are the most pronounced, and are characterized by irregularly distributed grooves, while the mass loss occurs through the detachment of larger fragments. Abrasive grains embedded in the specimen’s surface can also be observed. The Creusabro 4800 steel, despite significant plastic deformation and spalling, shows a more favorable structure due to the directional orientation of the furrows, i.e., in the direction parallel to the abrasive flow. The abrasive wear of the Creusabro 8000 steel is characterized by the occurrence of wear debris and furrows, with some localized tearing of small material fragments. In the case of TBL PLUS, the primary wear mechanism is the formation of wear debris. Moreover, the grooves are arranged parallel to the direction of the abrasive flow. In contrast, microcutting is the dominant phenomenon in XAR 600, but it should be noted that slight plastic deformation also occurs, which is the result of the presence of bainite in the microstructure—a structure that is more ductile than martensite. When compared to the TBL PLUS and XAR 600 steels, the 38GSA steel in the as-hardened state shows significant wear changes in the form of irregularly arranged furrows, the detachment of larger fragments of material and wear debris.

The above observations are confirmed by microphotographs, which show the cross sections of the tested surfaces (Figure 11). In the case of the 38GSA (as-normalized condition) and the Creusabro 4800 steels, furrows can be observed, in turn causing significant changes in the profile height. In comparison, only a slight plastic deformation is present in the microstructure of Creusabro 8000, while locally narrow cutting pits can also be observed. In the case of the TBL PLUS steel, the surface is relatively smoothed. In particular, microstructural changes are observed, which are manifested by the decomposition of martensite and the formation of carbides. In the case of XAR 600, surface roughness and sharp edges are characteristic, verifying the main mechanism of wear by microcutting. The wear outline profile of the 38GSA steel in the as-hardened condition also shows features of microploughing, microcutting and the tearing of material fragments.

Microscopic observations of the sub-surface microstructures of the cross-sections taken longitudinal to the direction of the abrasive action (Figure 12) provide more detailed information on the wear processes of the studied steels. In the case of the Creusabro, TBL PLUS and 38GSA (both heat treatment states) steels, there is an observed distortion of the crystallographic structure (texture) and the arrangement of martensite blocks in accordance with the direction of testing. These changes extend approximately 5 µm into the material in all of these materials and are indicative of the strengthening of the sub-surface layer by work hardening. In comparison, in the case of XAR 600, the above phenomenon occurs to a minimal extent and concerns the bainitic phase. As such, fragments of the material are cut out and there is no change in the structure. It should be noted that the chemical composition of the XAR 600 steel, when compared to that of the TBL PLUS steel, is characterized by a higher content of chromium, molybdenum and nickel. The carbon content is similar, and silicon and boron are present in nearly equal amounts. Morphologically, both materials show a microstructure consisting of lath martensite, and the grain size of the former austenite in these steels is analogous. On this basis, it can be concluded that the above alloying additives cause significant strengthening of the steel and thus lowering of the plastic properties, so that tribological wear occurs only through microcutting.

The above observations may also justify the different indices of the steels in question when they are tested in abrasive soil masses. According to Figure 1, excluding 38GSA steel in the as-normalized state, only the resistance of XAR 600 steel decreases with the change of light soil to medium and heavy ones. In this case, the above phenomenon can be explained by the tendency of the other materials to deform, and thus create a strengthened sub-surface layer. This is an obstacle to its penetration through compact, clay soils. Moreover, the nature of wear observed in TBL PLUS and Creusabro 8000 steels in the form of microploughing explains the obtained similar values of abrasion resistance when compared to higher-grade steels.

## 4. Conclusions

The results presented in this paper provide a comparative analysis of the effect of the microstructural properties and chemical composition on the abrasive wear resistance of the selected low-alloy, high-strength martensitic steels with a nominal hardness of 500–600 HBW. Based on the study, it can be concluded that:

− Creusabro steels can be classified as low- and medium-carbon steels, while TBL PLUS, XAR 600 and 38GSA are medium-carbon steels. The main hardenability-enhancing element in all the grades is manganese. It is also chromium, molybdenum and nickel in the Creusabro 8000 and XAR 600 steels, as well as silicon in the Creusabro 8000 and 38GSA steels. Boron is also a micro-additive in the TBL PLUS and XAR 600 steels.
− The microstructures of the TBL PLUS, XAR 600 and 38GSA (HT) steels consist mainly of lath martensite. Morphological differences include the presence of untempered martensite (TBL PLUS) and trace amounts of bainite (or Widmanstätten ferrite) at the grain boundaries of the prior austenite (XAR 600) due to the high content of aluminum (close to 0.1%wt.). 38GSA steel has the largest packet size. The different Creusabro steels also exhibit varied microstructural features, i.e., a structure of tempering sorbite in the case of the Creusabro 4800 steel and a martensitic-bainitic structure in the Creusabro 8000 steel.
− All the analyzed steels should be classified as fine-grained. The grain size of the prior austenite of the Creusabro 8000, TBL PLUS and XAR 600 steels is equal to 18.2–18.3 µm, while a larger size is characteristic of the Creusabro 4800 and 38GSA steels (d_av_ of 21.3 and 23.8 µm, respectively). As a result, the above variable that could affect the degree of wear may be excluded.
− The abrasive wear resistance of the Creusabro 8000, TBL PLUS, XAR 600 and 38GSA (HT) steels, determined in laboratory tests with the use of a T-07 device, is very similar to each other, with their coefficient of relative abrasion resistance k_bAV_ in relation to as-normalized C45 steel being 1.25–1.29. Due to the different hardness of the analyzed materials (non-linear relationship), the above phenomenon may be explained by the work-hardening of the sub-surface layer, as well as by the solid solution strengthening of the XAR 600 steel, which affects the different mechanisms of the tribological wear of the materials studied. In the case of the Creusabro 8000 and TBL PLUS steels, it is microploughing; in the XAR 600 steel, it comes mainly through microcutting; and the 38GSA steel shows complex features of wear by microploughing and the tearing of large material fragments. The work-hardening ability of lower-grade steel, that is, TBL PLUS and Creusabro 8000, allowed these steels to obtain similar wear indices as in the case of the 600 HBW grade steels.
− A microstructural analysis of the cross-sections of the surfaces subjected to abrasive wear tests confirmed the conclusions formulated above. It was shown that the microstructural features of the TBL PLUS steel are also altered by the decomposition of martensite. Moreover, in all the steels except XAR 600, a distortion of the sub-surface layer (texture) and an arrangement of martensite blocks in accordance with the direction of the tribological test occur. It should be noted that the carbon contents of the TBL PLUS and XAR 600 steels are similar to each other, and the chemical differences involve chromium, nickel and aluminum contents. On this basis, it can be concluded that the addition of the above alloying elements increases the strength of the material to such an extent that it exhibits low plastic properties, resulting in microcutting being the main wear mechanism.
− The above observations may also justify the different indices of the discussed steels when they are tested in abrasive soil masses. Based on the above results, soil grains cause the cutting out of the material in XAR 600 steel, resulting in a decrease in wear resistance in the presence of medium-to-heavy soil. For the other steels, the deformed sub-surface layer may act as a barrier to its penetration by the soil, in turn resulting in an increase in wear resistance.

## Figures and Tables

**Table 1:** The chemical compositions (%wt.) and hardness measurements with average standard deviation of the tested types of steels. PD—producer’s data, OR—own results [22,23,24]. In grey, there are marked elements which are further considered.

	Creusabro 4800		Creusabro 8000		TBL PLUS		XAR 600		38GSA	
	PD	OR	PD	OR	PD	OR	PD	OR	PD	OR
C	≤0.20	0.20	≤0.28	0.27	0.32–0.38	0.34	≤0.40	0.37	0.34–0.32	0.38
Mn	≤1.60	1.49	≤1.60	1.28	1.10–1.40	1.25	≤1.50	0.85	0.34–0.32	0.97
Si	-	0.35	-	0.70	≤0.40	0.21	≤0.80	0.19	0.80–1.10	0.90
P	≤0.018	0.012	≤0.018	0.012	≤0.020	0.012	≤0.025	0.014	0.035	0.011
S	≤0.005	0.004	≤0.005	0.002	≤0.025	0.010	≤0.010	0.001	0.040	0.007
Cr	≤1.90	1.45	≤1.60	0.68	≤0.50	0.25	≤1.50	0.83	0.30	0.05
Ni	≤1.00	0.30	≤1.00	0.29	-	0.08	≤1.50	1.21	0.30	0.08
Mo	≤0.40	0.16	≤0.40	0.23	-	0.03	≤0.50	0.15	-	0.02
B	-	-	-	0.0024	≤0.0040	0.0025	≤0.005	0.0021	-	-
Cu	-	0.23	-	0.20	-	0.08	-	0.03	0.30	0.25
Al	-	0.030	-	0.030	-	0.040	-	0.095	0.02–0.06	0.020
Ti	-	0.050	-	0.020	-	0.040	-	0.003	0.02–0.06	0.002
HBW	340–400	373 ± 14	430–500	463 ± 7	≤560	520 ± 6	≥550	555 ± 10	440	548 ± 8

**Table 2:** Mass loss of the tested types of steels (g) with standard deviation value.

No.	38 GSA (N)	Creusabro 4800	Creusabro 8000	TBL PLUS	38GSA (HT)	XAR 600
average	0.2533	0.2127	0.1837	0.1830	0.1781	0.1805
	±0.013	±0.006	±0.010	±0.007	±0.010	±0.008
